# ‘Old wine in a new bottle’: conceptualization of biodiversity offsets among environmental practitioners in Uganda

**DOI:** 10.1007/s00267-022-01639-2

**Published:** 2022-04-08

**Authors:** Ritah Kigonya

**Affiliations:** Department of Geography, Norwegian University of Science and Technology, Trondheim, Norway

**Keywords:** Biodiversity offsets, Conceptualizing, Compensations, Mitigation, Environmental practitioners, Uganda

## Abstract

Biodiversity offsets are increasingly adopted to mitigate the negative impacts of development activities on biodiversity. However, in practice, there are inconsistencies in how biodiversity offsets are understood and implemented. Based on interviews with environmental practitioners, the study sought to explore the conceptual understanding of biodiversity offsets among personnel involved in the design and implementation of offset schemes in Uganda. The study employed a ‘technical use analysis’ to seek personal interpretation and operationalization of the concept of biodiversity offsets. The results revealed that the concept tends to be simplified and adjusted to individual, project, and country contexts. The respondents had varied perceptions of biodiversity offsets in practice as compared to the theoretical concept. Biodiversity offsets were classified under five terms: trade-offs, payments, substitutes, compensations, and mitigation measures. The terms were derived from perceived inability of the measure to attain no net loss, and similarities of biodiversity components and services across impact and offset sites. Biodiversity offsets were thus considered no different from ordinary environmental conservation measures, contributing nothing unique to the conservation agenda. The study concludes that widespread implementation of biodiversity offsets under prevailing perceptions will escalate biodiversity loss. The study recommends emphasis on attaining no net loss through implementing outcome-based offsets as opposed to purpose-based offsets, that require delivering of ‘no net loss’ gains prior to projects being considered biodiversity offsets.

## Introduction

Biodiversity offsets (hereafter, BOs) are conservation activities implemented to compensate for the residual adverse impacts on biodiversity caused by development projects in one place by creating “equivalent gains else-where” (Apostolopoulou and Adams 2017). The goal of BOs is to achieve ‘no net loss’ (hereafter NNL) and preferably a net gain (hereafter NG) of biodiversity with respect to species composition, habitat structure, ecosystem function and people’s use and cultural values associated with biodiversity (BBOP 2009a). BOs are implemented in a number of forms across the globe, under names such as compensatory mitigation, mitigation banking, habitat banking, species banking, conservation banking, biodiversity banking, and wetland mitigation (Lapeyre et al. 2015). The growing popularity of biodiversity offsetting (Bull and Strange 2018) has led to the development of a widely recognized definition and best-practice guidelines (BBOP 2009b). Despite this, there are many inconsistencies and variations in how BOs and its encompassing concepts are interpreted and consequently implemented by different stakeholders (Brownlie and Botha 2009; Bull et al. 2013; Maron et al. 2018). However, there are no empirical studies exploring these various interpretations.

BOs analysis tends to be based on the assumption that there is a commonly agreed and consistent interpretation and use of the concept, but this may not be the case in practice. For example, Bull and Strange (2018) identified only one biodiversity offset project in Uganda, the Kalagala biodiversity offset, in their mapping of the global implementation of BOs. In their study, all projects aiming at completely compensating for all environmental impacts (to achieve ‘no net loss’) resulting from a development activity were classified as BOs. These can be considered purpose-based BOs, as their classification is based on the presence of clearly defined objectives aimed at achieving NNL. This is irrespective of actual attainment of the stipulated NNL goals, that would constitute what would be considered outcome-based BOs. However, there exists a wide range of conservation compensatory initiatives in Uganda that are locally considered to be BOs (Nabanyumya et al. 2017) despite the lack of the NNL goal. An investigation of BO practices in Brazil (Souza and Sánchez 2018) revealed that key legislation governing BOs do not require demonstrating NNL. In South Africa, BOs implementation focuses on maintaining a total amount of natural habitat as opposed to achieving NNL in the strictest sense (Brownlie and Botha 2009; Brownlie et al. 2017). Following the NNL guidance for the European Union Commission (Tucker et al. 2020), countries in the European Union (EU) are expected to consider NNL in relation to the country’s national formal targets for biodiversity conservation (Simmonds et al. 2020). This indicates that there are different interpretations of what BOs are about. This ambiguity may not be so visible or clarified in project and policy documents as recommended in academic studies (Maron et al. 2018).

Little is known about the actual conceptual understanding of BOs among those responsible for implementing the policies and projects, and how these perspectives influence BOs implementation. According to Short (1991, p. 29), “for research to be applicable in solving contemporary problems and achieving our purposes, it must be based on adequate interpretations of concepts, not on stipulative definitions which may fail to capture important aspects of their meaning. Research based on stipulated definitions runs the risk of being trivial or irrelevant.” Analyzing the understanding of a concept among different stakeholders also creates awareness of the possible perspectives that individuals may have and difficulties they may face in its implementation (Osborne and Gilbert 1980). The study sought to explore the conceptual understanding of BOs among personnel involved in the implementation of the measure schemes in Uganda. The study’s aim is addressed through answering three questions; (i) What are the perceptions held about BOs and their implementation? (ii) what are the reasons for the different perceptions? (iii) what are the resultant consequences of the various perceptions on BOs implementation?

## Background

### Biodiversity offsets and No Net Loss

**Fig. 1:**
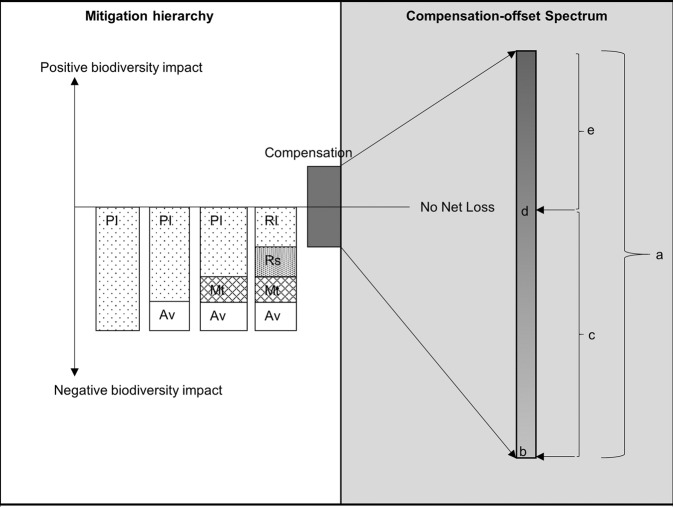
The mitigation hierarchy and compensation-offset spectrum (Source BBOP 2012. 2018). a Compensation spectrum, b No compensation, c Some investment in conservation but not quantified to balance the impacts/compensations with partial compliance with BBOP standards; d Offset with no net loss (meeting BBOP standards); e Offset with a net gain (meeting BBOP standards). PI predicted impacts, Av avoidance, Mt mitigation, Rs restoration, RI residual impacts.

BOs are commonly viewed as measures that create comparable and/or additional biodiversity gains to compensate for developmental impacts (Bull et al. 2013). According to the Business and Biodiversity Offset programme (BBOP) (2012), BOs lie on the compensation spectrum but only achieved when the compensation gains are equivalent to the biodiversity losses, resulting into a NNL (Figure 1). Therefore, BOs are a subset of compensations. Compensation measures can be framed as substitutions, tradeoffs or barters (Pope et al. 2021). When the biodiversity benefits obtained at the offset site are deemed greater than the biodiversity losses at the impact site, a NG is achieved. Biodiversity offset projects that have fallen short of attaining the NNL goal are still classified under BOs (Bull and Strange 2018). However, Maron et al. (2016, p. 496) recommends that projects that fail to fully attain biodiversity offset standards should be defined as ‘compensation initiatives’ rather than BOs. The recognition of projects that do not achieve NNL as BOs could inherently change the perception of what the acceptable standards of BOs are and hinder appropriate implementation of the measure amongst practitioners. The need for conceptual clarity is also emphasized by Pope et al. (2021) as an aid to appropriate implementation that can consequently lead to full compensation for biodiversity loss.

To operationalize BOs, nature components (biodiversity) and/or their functions (services) should be characterized and metrices created to measure their value. These metrics are derived from various biodiversity/ecosystem attributes or surrogates (e.g., certain habitat variables such as size, measures of disturbance) and is the ‘currency’ that allows loss at one site to be compensated for at another site. Since it is impossible to fully re-create the biodiversity loss at one site, one combination of attributes at the site of loss can be compensated by another combination of attributes of the same ‘value’ at the site of compensation. This applies to both biodiversity and ecosystems of the same kind and those in which the offset gains are of features different from those impacted, thus aiding the implementation of both ‘like for like’ and ‘out of kind’ BOs respectively (Bull et al. 2015). However, ‘like for like’ exchanges are preferred, and where ‘out of kind’ exchanges are to be carried out, biodiversity considered of greater or better conservation value is recommended as a compensation, a practice known as ‘trading up’ (BBOP 2012).

Largely, the efficacy of BOs is questioned. There is insufficient evidence from the field that NNL/NG can be achieved, with several assessments reporting that offsets ultimately lead to biodiversity loss (Maron et al. 2012; Curran et al. 2014; Thorn et al. 2018). The impediments are created by several challenges including: lack of a single metric that can be used to accurately measure losses and gains in biodiversity; defining requirements for demonstrating achievement of NNL; demonstrating equivalence between biodiversity components that differ in type, location, time or ecological context; uncertainty of how long development impacts will last and whether the offset will last for an equivalent time period; and uncertainty of whether the offset benefits will be realized (Bull et al. 2013). Considerations regarding peoples’ use and cultural values associated to biodiversity have also been reported absent in biodiversity offset discussions (Apostolopoulou and Adams 2017) and implementation (Bidaud et al. 2018). According to Griffiths et al. (2019) there is limited knowledge on how to ensure these are fully compensated.

The limitations of the BOs measure have instilled various perceptions among stakeholders. BOs have been deemed technically unrealistic (Walker et al. 2009) and largely implemented without appropriate scientific backing (Burgin 2008). To some, BOs cannot achieve NNL in practice (Hayes and Morrison-Saunders 2007; Souza and Sánchez 2018). The concept of NNL is also considered flawed, without practical realizations and merely a buzz phrase^1^ (Burgin 2008; Walker et al. 2009; Maron et al. 2018). To increase chances of attaining the NNL outcome, Gardner et al. (2013) recommend strict adherence and implementation of the measures along the mitigation hierarchy, which enables avoidance of un-offsetable impacts and minimization of residual impacts.

### Biodiversity offsetting in Uganda

Biodiversity offsetting is a relatively new conservation measure in Uganda. The country’s first encounter with the measure was when the Government of Uganda (GoU) was required to implement the Kalagala offset. This was a condition to attain a development loan from the World Bank (WB) in order to implement the Bujagali Hydropower project (IPN 2002, 2009; World Bank 2007). Two more BOs were implemented by the Uganda Wildlife Authority (UWA) and the National Forest Authority (NFA) to offset clearance of forest vegetation while establishing the Mbarara-Nkenda and Kawanda-Masaka electricity transmission lines respectively. These were also funded by the World Bank to facilitate implementation of the Environmental and Social Performance Standard 6 on Biodiversity Conservation and Sustainable Management of Living Natural Resources (IFC 2012).

Prior to the implementation of the BOs, it was realized that the environmental institutions in Uganda lacked the capacity to plan, implement and manage the BOs (IPN 2008). Therefore, for most of the staff, implementing the BOs was an experience best characterized as ‘learning by doing’. In 2016, the Conservation, Impact Mitigation and Biodiversity Offsets in Africa (COMBO) project was launched. This aimed at building institutional capacity in designing and implementing the mitigation hierarchy and biodiversity offsetting through trainings and workshops, and by sharing lessons learnt (COMBO 2016). The project supported the development of the Uganda Biodiversity and Social Offset Strategy (MWE 2019). Its goal is to suggest approaches, institutional arrangements and enhance the technical capacity that are necessary to implement the mitigation hierarchy and reconcile economic development with specific national targets for conservation of biodiversity in Uganda (MWE 2019). In addition, the project supported the amendment of the National Environmental Act to include provisions for implementing BOs (NEMA 2019). The official definition as laid out in the Act stipulates that BOs are measurable conservation outcomes resulting from actions designed to compensate for significant residual adverse biodiversity impacts arising from project development and persisting after appropriate prevention and mitigation measures have been implemented.

## Methodology

To ascertain how the environmental practitioners in Uganda conceptualize BOs, the study employed a ‘concept interpretation inquiry’. This inquiry seeks an adequate concrete interpretation of a concept (Short 1991). Semi-structured interviews were carried out with 18 environmental practitioners from environmental and resource management institutions, academic institutions, electricity transmission company, as well as independent environmental consultants (Table 1).

**Table 1:** Number of respondents from the different environmental management institutions

Identification (ID)	Institution	Number of respondents
NRMI	National resource management Institution	
	National Forestry Authority (NFA)	1
	Uganda Wildlife Authority (UWA)	1
	National Environment Management Authority (NEMA)	1
Mn	Ministry	
	Ministry of Water and Environment (MWE)	2
	Ministry of Agriculture, Animal Industry and Fisheries (MAAIF)	1
	Ministry of Energy and Mineral Development (MEMD)	1
NGO	Non-governmental organizations	
	Wildlife Conservation Society (WCS)	1
	Africa Institute for Energy Governance (AFIEGO)	1
	National Association of Professional Environmentalists (NAPE)	2
	Ecological Trends Alliance and	1
	Albertine Rift Conservation Society Uganda (ARCOS)	1
	World Animal Protection	1
Com	Company	
	Uganda Electricity Transmission Company Limited (UETCL)	1
Un	University	
	Makerere University	1
Con	Consultants	
	Consultants	2

The respondents were selected on the basis that they had directly (at project level) or indirectly (contributing to policy discussions) engaged in the formulation and/or implementation of BO projects and policies in the country. The respondents were obtained through both stratified random sampling and snowball sampling. The first respondents were obtained through stratified random sampling from a list of participants who took part in the formulation of BOs regulatory framework in Uganda. These engaged in consultations and discussions that led to the formulation of the National Biodiversity and Social Offset Strategy, and the amendment of the National Environment Management Act to incorporate provisions for biodiversity offset implementation in the country. The strata considered were the organizations they belonged to. In case the participant selected randomly was not available, another would be selected randomly from the same strata. In some cases, the participants referred other individuals to be interviewed, leading to snowball sampling.

I carried out unstructured, in-depth interviews (Ritchie and Lewis 2003) in which the respondents were given leeway to express their knowledge, experiences, and views on BOs. Prior to the interviews, the respondents were provided a research information document to create awareness about the theme to be discussed and how their data was going to be handled, after which their consent to be interviewed and use their data was obtained. Among the information sought for included the respondent’s personal understanding of what BOs mean or entail; perspectives considered while implementing biodiversity offsets; activities the term is used to characterize; and conditions thought to satisfy BOs (see interview protocol in Supplementary Material).

The definitions and/or explanations of what BOs are as provided by the respondents were subjected to conceptual analysis to identify the characteristics attributed to BOs measure. Conceptual analysis is a ‘mode of analysis by which we come to a sound understanding of the ordinary meaning of a concept’ (Short 1991). Since the study focused on ascertaining how the users or implementers of BOs understand and operationalize the concept, a technical use analysis was used. A technical use analysis is a process by which researchers seek to determine how a term is used by experts in the field (Kahn and Zeidler 2017). The analysis provides an account of a range of diverse and sometimes conflicting meanings a concept has among the technical users (Short 1991; Cox and Graham 2009). One way to ascertain an individual’s knowledge of a concept is through their ability to properly categorize instances not previously encountered, as instances or non-instances of a particular concept (Henmon 1971, p. 6). The data collected was organized, managed, and analyzed in NVivo software package. Recordings were uploaded into and transcribed by the researcher within the software package. The software allows for coding, a process of abstracting text from the interview responses and categorizing it under themes (Joffe and Yardley 2004). Generation of nodes was through inductive coding (data-driven), based on key characteristics or attributes of BOs as stipulated by the respondents. Based on the interviewee responses, major topics (Super codes) were first created, under which subtopics were created. The major topics formulate the major sections in the result section, while the subtopics formulate the subsections. The subcodes constitute terms that were explicitly used by the interviewees to describe their perceptions of the BO concept. During the analysis and classification of the information into codes, the researcher revisited the description of every code while examining the patterns across the statements in each transcript. For consistency in the coding, the process was completely carried out by the researcher. The recognized definition of BOs in literature served as a hypothetical meaning of the concept. The coding criteria is provided in Table 2.

**Table 2:** Inductive codebook developed on the conceptualization of BOs among environmental practitioners

Code	Code: explanation
Super codes	
Contextualizing	Expressions of simplifying or contextualizing the concept
Framing	Perception with respect to the manner the concept was constructed
Conceptualization	Perceptions of what the concept entailed
Inability to offset nature	Perceptions in relation to why offsets cannot attain NNL
Consequences	Implementation strategies that have resulted from perceptions held by the practitioners.
Subcodes within conceptualization super code	
Tradeoffs	Involve the loss of a set of biodiversity components in exchange for socio-economic benefits
Payment	Offset sites are a form of payment (biodiversity components or ecosystems) provided for loss of biodiversity components, irrespective of similarity in components lost and gained.
Substitute	Offsets are considered as forms of substitutes for biodiversity components lost. The impact and offset sites should have similar biodiversity components.
Compensations	Offsets are compensations that serve the same purpose as the biodiversity components or ecosystems lost
Mitigation measures	Offsets are mitigation measures, further minimizing the residual environmental impacts. The measures partially compensate for the residual impacts.

Research findings are potentially influenced by the identity of researcher and the participants, via our perceptions of others and the ways in which we expect others will perceive us (Bourke 2014). According to Osborne and Gilbert (1980), if an individual was to explain the meaning of a concept, their explanation would be influenced by the individual’s perception of the audience and what the audience required. While describing the meaning of BOs, the respondents took into consideration the fact that I was a scholar, knowledgeable of the formal definition of BOs and its implementation. The respondents could have explained the meaning of the concept differently if it was another interviewer with no prior knowledge of the concept, or if the question posed to them was not requiring them to provide their personal understanding of the concept.

## Results

Four themes appeared in the environmental practitioner interviews: Contextualizing, framing, conceptualization, and the inability to offset nature.

### Contextualizing

When requested to provide their understanding of the BOs concept, the respondents often started by expressing the need to simplify or contextualize the concept. Through contextualizing, the respondents provided an understanding of the concept within an individual, project, or country context. Among the typical phrases used among five respondents including a wildlife campaign manager in an NGO (NGO1); a chief executive officer of an NGO (NGO2); a project manager of an offset project (NGO3); a consultant in environmental/social planning and management (Con1); and a forest utilization specialist in a national resource management institution (NRMI1) are provided in Table 3 (italicized):

**Table 3:** Simplifying or contextualizing statements used by respondents while explaining their understanding of BOs

Respondent	Interview quotation
NGO1	…if you put it in *simple terms*… *For me* that is how I would put it.
NGO2	…the conceptualization of what is an offset in *as far as Uganda* is concerned…
NGO3	…the conceptualization of what is an offset in *as far as Uganda* is concerned…
Con1	I will basically want to look at it *in terms of what we do*… I think that is *my simplified* way of understanding it. So, I am looking at it basically from the *project perspective*
NRMI1	…according *to me*, and actually according *to us as an organization*…

Despite the simplification, some respondents reported knowledge and awareness of the formal definition of the concept. As one of the technical advisers in forestry, environmental and ecosystem management, put it:

… we do know the actual concept, according to BBOP, and we recognize it, but when it comes to implementing, we compromise. (Con2)

By using the word compromise, the respondent recognized that there are aspects within the formal guidelines of BOs implementation that are deliberately left out, or not considered, or not implemented or modified while implementing offsets in Uganda. An executive director of an environmental NGO pointed out some of the aspects that are compromised when he reported what constitutes a strict BOs scenario:

There should be an idea of NNL in terms of numbers, content, though it might take a number of years for the composition in the offset site to be similar to that in the development site. Idea of continuity has got to be there, which can be attained through strengthened management. In the strict sense, there has got to be continuity, similarity, additionality… If you follow that through, then you will offset. (NGO4)

NGO3 explicitly mentioned setting aside the definition by BBOP for one deemed applicable to Uganda’s context (response in Table 3). The respondents depicted a lack of full operationalization of the theoretical concept in their working contexts. The simplification and contextualization of the concept was geared towards respondent’s engagement in operationalizing the concept in given contexts.

### Framing

Some of the respondents do not consider that the term ‘biodiversity offsets’ reflects the totality of what it intends to describe. While discussing the concept, a program director in an environmental NGO, stated that:

I always avoid to say only biodiversity offset because then that’s where you start by putting aside social offsets. (NGO5)

NGO5 argued that the term biodiversity offsets does not explicitly take into consideration compensation of benefits (services) the society obtains from biodiversity, here termed ‘social offsets.’ In other words, the term puts emphasis on the biodiversity components while taking for granted the human nature interactions. To bring out the totality of what the measure is meant to address, NRMI1, suggested inclusion of the words ‘social’ and ‘economic’

It should be biodiversity and social offset. The title will be long. But even the word economic should appear somewhere: Biodiversity social economic offset. It (biodiversity offset) is not as explicit. It is not telling us that we are really dependent on it (biodiversity). (NRMI1)

To NRMI1, BOs as a term does not depict the human dependency on nature and proposes the incorporation of the words ‘social’ and ‘economic’. This would bring out a wholistic picture of the importance of biodiversity and human dependency on it, as well as the need to take this into consideration while implementing BOs. By ‘economic’, NRMI1 was referring to the economic benefits the communities obtain from engaging in biodiversity-based livelihoods. However, compensation of biodiversity-based livelihoods is a component inherent in social offsets.

### Conceptualization of biodiversity offsets

On a conceptual level, the respondents framed BOs as trade-offs, payments, substitutes, compensation measures and mitigation measures.

#### Biodiversity offsets as a trade-off

One of the respondents considered BOs a trade-off, as any attempt to duplicate already existing ecological systems in another locality at a given time cannot be fully realized. In NRMI1’swords:

(Biodiversity offsetting) is the intention to duplicate a system… they are trade-offs we have made to get the development we desire. Any attempt to create a biodiversity offset is just an effort to as much as possible, maybe to any effort there was, but cannot take care of the insufficiencies that we had either predicted that would be enough to drive economic development in this country…. So, there is a loss (in biodiversity) and yet our new thinking is that in biodiversity and social offsets, there should not be any net loss.

NRMI1 notes that no matter the efforts invested; ecological systems cannot be duplicated. Therefore, the system components that are to be lost during the establishment of the development activities cannot be fully replicated at the offset site. NRMI1 reechoed concerns shared by Daly (1994); that nature and nature-produced services and resources are not replicable. The specialist argued that BOs will inevitably result in loss of some biodiversity components. Therefore, those implementing BOs forego some ecological benefits in exchange for some social economic benefits, which is a tradeoff (Wright and Burns 2007; Brownlie et al. 2013).

#### Biodiversity offsets as a payment

Biodiversity offsets were also considered a form of payment for the negative impacts caused on biodiversity. According to NGO3:

These are some measures that you take to, like pay for the negative impacts that you will cause on a particular site for the project that you are implementing. And my relation to that is depending on the area that you are impacting, because you may not (in Uganda) get like for like. Because literally our landscape is really partitioned up. So, if you can get something to work on and improve the value…because you will not get, I might not get like for like. I may be destroying forested land and I have to work on a woodland.

Due to the unlikeliness to get a similar ecosystem to preserve or restore as a compensation for one affected, the respondent considers the offset area as a form of payment. Something that has been provided in exchange for what has been affected (irrespective of similarity in composition). The respondent recognizes that fragmentation of the landscape and ecosystems hinders attainment of similar impact and offset sites. In that case, the impacted and offset sites can serve totally different roles and provide different benefits. What the respondent explains here is similar to what Walker et al (2009) termed biodiversity bartering or exchanging. There is no equivalence in what is lost and gained, but there is negotiation over goods and services that are restored or conserved in place of what is lost.

#### Biodiversity offsets as a substitute

To some respondents, BOs were considered as areas set aside as substitutes to secure environmental components impacted in other areas due to the establishment of a development activity. The substitute sites should constitute similar biodiversity components, just as NGO2, and the head of the health, safety and environmental unit of a ministry (Mn1) stated:

…if you know you cannot avoid negative impacts or you cannot mitigate … proceed to do these things (developments) and these are the impacts, but also have an idea of how you can make sure that those things you have lost here, you can actually maintain them in another place… another area that you try to use as an alternative to this one. (NGO2)

…. So, in order to replace for what has been lost, you go, maybe get an area which is also a central forest reserve, or buy a particular piece of land, then you should be able to replace what was lost the other side. (Mn1)

To NGO2 and Mn1 the components lost in the impact site should be secured or protected or enhanced in another site, thus in-kind substitutes. Con1 also stated:

It (BOs) is synonymous with an area that will have to be set aside specifically to make sure that where a project has had impacts on biodiversity in one way or the other, you create another area specifically to make sure that you generate similar or better biodiversity as an offset to what you will impact. (Con1)

According to Co1, the biodiversity in the area set aside as a substitute should be similar (of the same kind) to that lost or deemed of greater conservation value (or priority) compared to the one lost, an action referred to in section 2 as ‘trading up’. Trading up is an option taken mainly when the environmental impacts are not offsetable (IUCN 2014). However, the choice to either strictly adhere to like-for-like or trading up depends on the conservation status and value of species impacted.

#### Biodiversity offsets as a compensation

For others, BOs were compensations to counteract lost biodiversity components and/ or services as reported by a ministerial environmental specialist (Mn2), a lecturer at a University (Un1) and the wildlife campaign manager (NGO1) shown in Table 4.

**Table 4:** Quotations of practitioners referring to BOs as compensations

Respondent	Interview quotation
Mn2	We create some conditions elsewhere that would compensate for the destruction, that residual destruction.
Un1	These (BOs) are compensation mechanisms for loss of biodiversity resources and the services that these resources offer beyond just the biodiversity itself.
NGO1	I think it’s kind of compensating for something that you have lost here; it’s compensated for elsewhere. So that ultimately, you don’t experience that you have lost anything. It’s just like say…. if you take away this dress, … I should still be dressed. I shouldn’t remain naked. So, you might take away a grey dress for some reason and then you sell the dress with a red dress. Meaning that ultimately, I am dressed. Its compensating for something that has been taken away from you and is replaced by something that would still serve the same purpose without feeling that you lost anything.

To NGO1, the components at the compensation site should be able to serve the same purpose, as those lost at the impact site. This is irrespective of the kind of biodiversity, as long as the ultimate service being provided is more or less the same. This would ideally take into consideration both in-kind and out-of-kind biodiversity offsetting. Un1 put emphasis on enhancing lost biodiversity services in the process of compensating for lost biodiversity. However, he did not stipulate whether the biodiversity should be of the same kind or not.

All the above classifications refer to BOs measures as compensations of different forms. Trade-offs, payments and substitutes are compensations (Pope et al. 2021) that involve exchange of one component or service with another that either serves the same or different purpose. Respondents used different terms to provide a personal view of what the compensation entailed. The similarity or deference in biodiversity components and services lost and gained played a role in defining the type of compensation it is.

#### Biodiversity offsets as a mitigation measure

Biodiversity offsets were also simply considered a *mitigation measure*. A planning and Environmental Impact Assessment (EIA) Officer (NRMI2), attributed this classification to the presence of the conservation measure on the mitigation hierarchy:

It is on the mitigation hierarchy. So, it is a mitigation. (NRMI2)

To NRMI2, situating BOs on the mitigation hierarchy makes it a mitigation measure aimed at limiting negative impacts of development activities on biodiversity. A senior internal monitoring and evaluation officer of a resource management institution (NRMI3) also classified BOs among the mitigation measures on the hierarchy:

An offset comes when we really see that the other three mitigation hierarchy; avoidance, mitigation and restoration, have now failed. (NRMI3)

By saying “three mitigation hierarchy”, NRMI3 was referring to “the three stages on the mitigation hierarchy”. The first three stages constitute mitigation measures, are implemented at the site of development, by the developer to minimize the extent and magnitude of impacts. Implementation of the mitigation measures, is essential and a prerequisite for successful implementation of BOs (Gardner et al. 2013, p. 1258). However, the appearance of BOs as the last stage of the mitigation hierarchy, does not explicitly reveal that the measure is implemented offsite as a compensation measure for residual impacts after mitigation measures have been exhausted. A director of environment affairs (Mn3), a senior environmental officer (Mn4) and an environmental specialist (NRMI4) also considered BOs a tool for mitigating residual impacts (Table 5).

To NGO1, BOs are mitigation measures due to the presumed inability to fully compensate for residual impacts to attain NNL.

Our inability to understand the full range of nature interactions is a hindrance to our ability to compensate for nature…. Nature is very, very complex and no single person, I think, or science can aid us to understand the full range of interactions in nature…. So, for me BOs, I think they are about minimizing the loss and in terms of what people feel as the impact on their access rights for utilization, and then of course the entire ecosystem, so that the system continues to function. (NGO1)

NGO1 argues that humans do not comprehend nature and its relations to a level that can enable full compensation of environmental impacts. In that case, BOs are limited to minimizing biodiversity loss. Our inability to fully compensate for nature was further stressed by more respondents as discussed in the next section.

### Inability to offset nature

Almost all the respondents emphasized the inability to offset all kinds of biodiversity. An executive director (NGO6) and a chief executive officer (NGO2) of environmental NGOs elaborated:

When you talk about biodiversity actually, it is bio, it is life, and we are talking of many things connected and unconnected…. there are those that you cannot offset. (NGO6)

And of course, we still believe that, you know, sometimes it is not possible (to offset) because nature is nature, the things that are here can never be completely the same things on the other site. (NGO2)

The challenge of irreplaceability of some biodiversity and ecosystems that the respondents were pointing out has also been emphasized within conservation planning (Margules and Pressey 2000). In elaborating the inability to offset all biodiversity components, the respondents’ reflections mainly encompassed seeking for similarity in biological components across space and time. There was a clear lack of consideration of the use of surrogate metrices to calculate losses and gains in biodiversity to aid the achievement of NNL.

NGO2 partly attributed the inability to offset some components of nature to the specificity and uniqueness of biodiversity components in particular locations, with minimal potential of establishment and survival in other places. This view was also held by NGO6 who reported that:

And in any case, even if you take it (biodiversity components being offset) somewhere (else), it may not survive. For example, why do you think there are lions in one place and in another place, there are no lions? Why do you think there is a wetland here but then there is not a wetland up there? Nature created itself that with the help of God the creator that the wetlands are supposed to be in the valleys and then there is supposed to be a hill. So, if you want to shift this one and put it in another place, it will not fit. (NGO6)

According to NGO6, there are attributes specific to nature that require biodiversity components to exist in particular localities. An officer of forests and biodiversity at an NGO (NGO7) also expressed concerns in offsetting non-tangible benefits of nature:

If you had this wetland for example, its serving Lubowa (a place) and Nfufu (a place). And you want to put a factory here and then do offsetting, does it mean that the water that was getting stopped here is going to go to a place where you have offset? Bujagali falls were destroyed and there was an offset; Kalangala offset. Does it mean that the beauty that people in Jinja (location of Bujagali falls) used to enjoy will certainly come or shift the other end? (NGO7)

To NGO7, some components of nature and associated benefits (both tangible and non-tangible) cannot be shifted across space.

There were also concerns regarding constraints in time lags between occurrence of environmental impacts and attaining BOs benefits. According to NGO4:

Some of these ecosystems are constructed over thousands of years, hundreds of years. You can even say decades. But something that is going to take 50 years to be re-constructed, you can imagine the things you will have lost in the 50 years, before you get to the 50 years.

The respondent pointed out the inability to attain equivalence in the loses and gains before the BOs sites exist for years equivalent to those of impacted sites. This time lag challenge has also been pointed out by other scholars (Gibbons and Lindenmayer 2007; Bull et al. 2013) which led to criticizing BOs for falling short of the principle of intergenerational equity (Gibbons et al. 2018).

### Does it matter? Consequences of different interpretations

Given the expectation that offsets should ideally attain NNL, almost all the implemented BOs projects are considered offsets in name but not in their operationalization. As NGO4 pointed out:

If you move around, you will get examples where people went with the thinking of offsets, but eventually did something else. But in the records, they are offsets, examples of offsets

To NGO4, the projects commonly acquired their categorization as BOs at the initiation stage based on the stipulated aim (to offset biodiversity impacts), and despite failing to achieve the many and complex objectives of BOs, they remained categorized as BOs on record. In the case of Uganda, “offsetting is an umbrella term encompassing a variety of conservation measures aimed at compensating the negative impacts of a development project, but with no emphasis on achieving NNL. As NRMI2 and NGO5 noted:

…the offset can take different forms. It can be management, improving management, it can be like restoration offset… (NRMI2)

… to me, offsetting can mean anything of course within the acceptable boundaries of offsetting…. if you destroy a forest, planting trees elsewhere can be only one option. I mean it can be one option but not the only one. It can also be strengthening management of another forest really, to me. So, to me it’s acceptable but other people say no. (NGO5)

To NRMI2 and NGO5, any conservation measure can be an offset as long as the stakeholders have agreed to consider it so. NGO5 embraces the diversity in views regarding what can be acceptable as an offset. This widens the scope of projects that can be considered as offsets in the country, but at the same time, losing the core of what an offset is.

With the lack of a clear difference between offset measures in practice and other conservation measures, some respondents referred to BOs as simply a new term encompassing already existing conservation measures. NGO2 considered biodiversity offsets as a re-branding of already existing conservation practices:

When we talk of biodiversity offsets, it is just like when we talk about climate change, doing things to mitigate against the impacts of climate change. If you look at the interventions that people are currently implementing in the name of either being prepared to mitigate against impacts of climate change or to stop actually climate change from taking place, those are things which were done many many years ago…. The difference is they were called something different…. So, for me I think, there is nothing new. It is kind of, they call it “*old wine in a new bottle*”, something like that.

To NGO2 there is nothing novel about BOs in relation to other conservation practices. The NNL goal, which is the only output that uplifts ordinary conservation measures to an offset, is not attained. This perception that BOs bring something new or unique to the table (of conservation practice) is dismissed and BOs are rather at best seen as a rebranding of existing conservation measures.

**Table 5:** Quotations where BOs are considered a tool for mitigation environmental impacts

Respondent	Interview quotation
Mn3	I think biodiversity offsets is a tool that is used to mitigate the negative impacts that may arise from…. development activities in as far as they impact on the environment
Mn4	…. a kind of mitigation measure that is put in place to reduce the impacts of development of maybe an installation
NRMI4	…. like you do a development and then there are those impacts along the mitigation hierarchy, those ones which we can manage, mitigate, then the offsets come in as the last resort. Anything you do to mitigate the impact

## Discussion

The perceptions stakeholders have regarding a conservation measure are reported to influence their attitudes, acceptability and levels of support for the measure (Bennett 2016). In theory, understanding the perceptions of conservation stakeholders could help understand the responses of the stakeholders to existing policies or activities (Gelcich et al. 2005). Exploring the perceptions and opinions of those involved in management processes can also inform operational and political realities that may be missing in the academic literature (Hopkins et al. 2016) or from more standardized management effectiveness evaluations (Pyhälä et al. 2019). The study reveals that practitioner’s perceptions on BOs vary and are linked to individuals’ BO implementation experiences. The practitioners provided their opinions of BOs with reference to occurrences within the BO initiatives they participated in. Practitioners’ earlier experiences with BOs initiatives influenced their subsequent perception about the initiative (Fazey et al.^ 2006^). As reported by Cook et al. (2014), practitioners’ judgements are greatly based on day-to-day management experiences. According to the practitioners, operationalization of the different BOs initiatives involved compromising on some BO’s standards and principals, leaving out aspects that were deemed unattainable in given contexts. Therefore, different practitioners were exposed to different BO implementation strategies within the different BOs initiatives, ultimately acquiring different perceptions of the BO measure. Whereas modifications in the implementation of BOs with respect to prevailing social, economic, political, and ecological contexts is recommended (Gelcich et al. 2017), the study reveals that this can create a plurality in ways of understanding the concept, such as what is deemed acceptable and within the margins of a biodiversity offsetting measure.

Based on the constituent words, practitioners tend to associate BOs with offsetting only the biological components. It is common practice to associate concepts to the words that constitute them although the word meanings are typically less broad and more constrained than the corresponding concepts (Borghi and Binkofski 2014, p. 1). Exclusion of the word social from the word construct of the term is equated to exclusion of social compensations within BO implementation, and thus partly a reason for the absence of social compensations during BOs implementation in Uganda. There is growing criticism of the limited or lack of focus on social compensations among scholars (Maron et al. 2016; Gelcich et al. 2017; Bidaud et al. 2018; Griffiths et al. 2020). Offsets relocate nature away from people and cities, changing the ecosystem services they obtain in a local area (Kalliolevo et al. 2021). They ignore links between people and nature and thus portray nature as external to society (Apostolopoulou and Adams 2017). In the attempt to make BOs more inclusive of the social concerns, some policy documents have incorporated the word social; “Biodiversity and Social Offsets” (MWE 2019) or used the phrase ‘No net loss of people and biodiversity’ (Bull et al. 2018; Griffiths et al. 2019) in reference to biodiversity offsets. These modifications have been made to emphasize offsetting of people’s use and non-use values in addition to offsetting biodiversity. Modifying the term BOs to ‘biodiversity and social offsets’ could be necessary to draw attention to the social economic aspects of offsetting. However, this does not guarantee increased attention and focus on social compensations. These have in some instances been unapologetically left out of biodiversity offset guidelines and implementation as they are considered complex and incompatible with biodiversity metrics (Maron et al. 2016; Taherzadeh and Howley 2018). It is also assumed that incorporating wider ecological parameters into BOs implementation to improve ecological compensation would implicitly factor issues related to distributive and procedural justice into the offsetting equation, resulting into greater social compensation (Taherzadeh and Howley 2018). Though criticized, the lack of focus on social offsets is a fundamental construct of BOs as a measure focusing on enhancing biodiversity benefits with minimal consideration of resultant social losses. As reported by Apostolopoulou et al. (2018), at the core of BOs is the production of equivalent natures with profound implications for communities.

BOs implementation is perceived by the practitioners to encompass ordinary compensatory measures classified as trade-offs, payments, substitutes, compensations, and mitigation measures based on the resultant biodiversity benefits or environmental outputs. Although such framings of the concept has been used before (Pope et al. 2021), the practitioners used these terminologies to point out the implications of the various forms of compensatory measures implemented. BOs were classified as trade-offs when they permit loss of given biodiversity components and services in order to attain desired economic benefits or services; payments when conservation benefits mainly of a different kind are established in exchange for lost biodiversity components and services; Substitutes when sites constituting biodiversity components similar to the impacted sites are set aside as replacements for what has been lost due to development activities; Compensations when offset sites provide similar ecosystem services as those generated in the impact site; and mitigation measures when they minimize rather than fully compensate for biodiversity losses due to development activities. The implementation of BOs as an ordinary compensation measure portrays a disconnect between the intention of biodiversity offsetting in theory and their materialization in practice.

Although the practitioners classified BOs as forms of compensations with varying terminologies, the practitioners also reported theoretical understanding of BOs as initiatives implemented with an intention to completely compensate for residual environmental impacts of development activities (BBOP 2012). However, the practitioners doubted the ability of BOs to attain NNL on ground. Similar to Gardner et al.’s (2013) view, the practitioners considered the measure’s potential to attain NNL only theoretical. A similar perception was also held among environmental practitioners in Western Australia (Hayes and Morrison-Saunders 2007) and Brazil (Souza and Sánchez 2018). The practitioners attributed inability to attain NNL to several constraints including the inability to attain spaces with similar biodiversity as the impacted sites; inability to create similar spaces as those impacted due to location specific attributes; irreplaceability of some biodiversity components; and time lags between occurrence of environmental impacts and environmental benefits. These constraints have been echoed among the theoretical challenges of biodiversity offsetting (Bull et al. 2013; Gardner et al. 2013). According to the practitioners, equivalence in what is lost and gained cannot be attained, but rather, negotiations are made over what is restored or conserved in place of what has been lost. Biodiversity compensatory activities implemented during offsetting ultimately results into loss of some biodiversity components.

Biodiversity quantifying frameworks are increasingly formulated and used to demonstrate equivalence in biodiversity losses and gains, especially between different kinds of biodiversity. However, the use of biodiversity quantifying frameworks was not reported, discussed, or reflected upon by the practitioners. The partitioners instead envisioned strict fungibility during offsetting. According to Karlsson and Edvardsson Björnberg (2020, p. 4), the measure does not aim for strict fungibility, but rather, recognizes the inevitable loss of biodiversity components during biodiversity exploitation and aims at making up for the lost values as far as possible. This could depict a bias towards in-kind offsetting as the ideal form of biodiversity offsetting among the practitioners, or a lack of knowledge and capacity in using quantifying measures to operationalize the BOs measure. Besides, there is no established methodology to calculate biodiversity gains and losses at the project and country level. The BOs trainings and workshops that have been carried out in the country have not constituted topics relating to biodiversity quantification measures. There is thus a need to train practitioners in the use of biodiversity quantifying frameworks to improve design and implementation of BOs.

Practitioner’s perception that BOs cannot achieve NNL has resulted into exemption of and a laxity in incorporating NNL targets in biodiversity offset projects in Uganda. BOs in the country encompass any conservation measure agreed upon by stakeholders to be an offset. They have been classified based on the purpose; as measures to compensate for residual development impacts, rather than the outcome; as measures that have fully compensated for residual development impacts of the initiatives. Consequently, there is lack of a clear boundary between BOs measures and other mitigation and compensatory measures (Darbi et al. 2009). These findings are not exceptional for Uganda, as several BOs projects have also been reported to lack the NNL goal (Souza and Sánchez 2018; Weissgerber et al. 2019; Silva et al. 2019). According to Brownlie et al. (2013), implementation of BOs that do not fully compensate for residual biodiversity impacts is the most common scenario, and it is anticipated to continue that way. Limiting BOs success to the benefits it provides to the environment without efforts to attain NNL is considered inappropriate (Hayes and Morrison-Saunders 2007), and could escalate biodiversity loss. According to the practitioners, it is a rebranding of existing conservation measures. These can then be used to fulfil the donors’ requirements and expectations. Especially, since BOs implementation has been a pre-requisite for financing development projects in the country (IFC 2012). This rebranding has enhanced the use of ostensible offsets by developers to maneuver stringent policy requirements for biodiversity conservation (Bond et al. 2020). BOs can be considered a buzzword, which is currently used by environmentalists to attract financing of environmental conservation activities (Githiru et al. 2015; Deutz et al. 2020). For the developers, a license to pave way for more development activities (ten Kate et al. 2004). In that respect, it is a little more than ‘old wine in a new bottle’. Continued rebranding of existing conservation measures as BOs misinforms conservation practice and policy in relation to paving a way for reducing biodiversity loss. This results into fewer biodiversity offsets in practice as compared to what is stipulated (IUCN 2019). Taking stock of BOs calls for classification of compensatory projects as BOs after attainment of the NNL goal. This will require implementation of the conservation measures prior to establishment of the development activities, which are then classified as offsets upon approval of offsetting residual impacts of the development activities (Bekessy et al. 2010). There is a need to move beyond the purpose-based offsetting to outcome-based offsetting. Requirements for demonstrating NNL prior to BO classification will reduce implementation of partial compensations (Moilanen and Kotiaho 2018), as well as motivate practitioners to incorporate NNL goals in offset implementation. Strict adherence to the achievement of a NNL goal might also result into more adherence to avoidance than establishment of BOs that would turn out to be more expensive (Hayes and Morrison-Saunders 2007). This will contribute to reducing the occurrences of potentially avoidable biodiversity offset losses.

## Conclusion

Currently, BOs are widely adopted and integrated within national and institutional regulatory frameworks. Yet, there is no empirical evidence that explores the stakeholder perceptions about the measure and its implementation. The study sought to understand the perceptions of environmental practitioners in Uganda towards BOs, and how the perceptions have influenced BO implementation in the country. Based on personal experiences while implementing the measure, practitioners described BOs as either ordinary compensation measures classified as trade-offs, payments, substitutes and compensations, or environmental mitigation measures. These classifications were attributed to the perceived inability of the measure to attain NNL of biodiversity. In addition, there was lack of knowledge and awareness creation of biodiversity quantifying frameworks as measures for attaining equivalence in biodiversity losses and gains. According to practitioners in Uganda, the term BOs has been used to re-brand existing conservation measures that are implemented as compensations for residual development impacts, but without stipulating NNL goals. The study revealed that practitioner’s perceptions about potential outcomes of a conservation measure influences implementation practice of the measure. Practitioners are more likely to incorporate conservation targets deemed achievable and leave out those deemed unachievable during implementation of conservation initiatives. Consequently, some conservation measures retain their classification by name but lack the core attributes in practice. BOs in practice are not qualified by the *NNL outcome*, but rather *purpose* of the measure to offset residual development impacts. To minimize biodiversity losses, there is need to emphasize outcome-based offsets as opposed to purpose-based offsets. This requires assessment of projects for achievement of desired objectives or biodiversity benefits prior to branding them as BOs. BOs practitioners should be educated and trained to use biodiversity metrices to enable quantification of biodiversity losses and gains to facilitate achievement of NNL especially during out of kind offsetting. Incorporating the frameworks with the country’s BOs implementation strategies and guidelines could facilitate improved BO implementation.

## Supplementary information

Assessment by NSD

Interview protocol

## Data Availability

Data available on request from author.

## Footnotes

1 According to the Oxford dictionary means ‘an item of Jargon, that is fashionable at a particular time in a particular context’
