# Supplementary material for: ‘Old wine in a new bottle’: conceptualization of biodiversity offsets among environmental practitioners in Uganda
Source: Environ Manage. 2022 Apr 8;69(6):1202–16. doi: 10.1007/s00267-022-01639-2 (PMC9079017; doi:10.1007/s00267-022-01639-2)
Supplement: Supplementary file 1 — Assessment by NSD [file 267_2022_1639_MOESM1_ESM.pdf]

NSD NORSK SENTER FOR FORSKNINGSDATA

NSD's assessment

Project title

Enhancing Livelihood Outcomes in Biodiversity offset Schemes in Uganda's Oil and Gas Industry's CSR Strategies

Reference number

722406

Registered

27.06.2019 av Ritah Kigonya - ritah.kigonya@ntnu.no

Data controller (institution responsible for the project)

Norges teknisk-naturvitenskapelige universitet / Fakultet for samfunns- og utdanningsvitenskap (SU) / Institutt for geografi

Project leader (academic employee/supervisor or PhD candidate)

Ritah Kigonya, ritah.kigonya@ntnu.no, tlf: 73591877

Type of project

Research Project

Project period

10.08.2019 - 31.12.2022

Status

17.07.2019 - Assessed

Assessment (1)

17.07.2019 - Assessed

Our assessment is that the processing of personal data in this project will comply with data protection legislation, so long as it is carried out in accordance with what is documented in the Notification Form and attachments, dated 17.07.2019, as well as in correspondence with NSD. Everything is in place for the processing to begin.

NOTIFY CHANGES
If you intend to make changes to the processing of personal data in this project it may be necessary to notify NSD. This is done by updating the Notification Form. On our website we explain which changes must be

notified. Wait until you receive an answer from us before you carry out the changes.

TYPE OF DATA AND DURATION
The project will be processing special categories of personal data about ethnic origin, and general categories of personal data, until 31.12.2022. The data will then be stored internal to the data controller until 31.12.2027, to make room for possible follow-up studies, as well as verification for publications.

LEGAL BASIS
The project will gain consent from data subjects to process their personal data. We find that consent will meet the necessary requirements under art. 4 (11) and 7, in that it will be a freely given, specific, informed and unambiguous statement or action, which will be documented and can be withdrawn.

The legal basis for processing special categories of personal data is therefore explicit consent given by the data subject, cf. the General Data Protection Regulation art. 6.1 a), cf. art. 9.2 a), cf. the Personal Data Act § 10, cf. § 9 (2).

PRINCIPLES RELATING TO PROCESSING PERSONAL DATA
NSD finds that the planned processing of personal data will be in accordance with the principles under the General Data Protection Regulation regarding:

- lawfulness, fairness and transparency (art. 5.1 a), in that data subjects will receive sufficient information about the processing and will give their consent
- purpose limitation (art. 5.1 b), in that personal data will be collected for specified, explicit and legitimate purposes, and will not be processed for new, incompatible purposes
- data minimisation (art. 5.1 c), in that only personal data which are adequate, relevant and necessary for the purpose of the project will be processed
- storage limitation (art. 5.1 e), in that personal data will not be stored for longer than is necessary to fulfil the project’s purpose

THE RIGHTS OF DATA SUBJECTS
Data subjects will have the following rights in this project: transparency (art. 12), information (art. 13), access (art. 15), rectification (art. 16), erasure (art. 17), restriction of processing (art. 18), notification (art. 19), data portability (art. 20). These rights apply so long as the data subject can be identified in the collected data.

NSD finds that the information that will be given to data subjects about the processing of their personal data will meet the legal requirements for form and content, cf. art. 12.1 and art. 13.

We remind you that if a data subject contacts you about their rights, the data controller has a duty to reply within a month.

FOLLOW YOUR INSTITUTION’S GUIDELINES
NSD presupposes that the project will meet the requirements of accuracy (art. 5.1 d), integrity and confidentiality (art. 5.1 f) and security (art. 32) when processing personal data.

To ensure that these requirements are met you must follow your institution’s internal guidelines and/or consult with your institution (i.e. the institution responsible for the project).

FOLLOW-UP OF THE PROJECT
NSD will follow up the progress of the project underway (every other year) and at the planned end date in order to determine whether the processing of personal data has been concluded/is being carried out in accordance with what is documented.

Good luck with the project!

Contact person at NSD: Jørgen Wincentsen
Data Protection Services for Research: +47 55 58 21 17 (press 1)
