# Supplementary material for: ‘Old wine in a new bottle’: conceptualization of biodiversity offsets among environmental practitioners in Uganda
Source: Environ Manage. 2022 Apr 8;69(6):1202–16. doi: 10.1007/s00267-022-01639-2 (PMC9079017; doi:10.1007/s00267-022-01639-2)
Supplement: Supplementary file 2 — Interview protocol [file 267_2022_1639_MOESM2_ESM.pdf]

**'Old wine in a new bottle': Conceptualization of Biodiversity Offsets among environmental practitioners in Uganda**

Journal: Environmental management

Ritah Kigonya

Department of Geography, Norwegian University of Science and Technology, Edvard Bulls Veg 1, 7491, Trondheim, Norway

Email: ritah.kigonya@ntnu.no

Supplementary material 1: Interview Protocol (constituting Topic 1 from which data for the paper was generated from)

## Introduction

The interviews are kicked off with appreciating the respondents for voluntarily accepting and creating time to take part in the interview.

Informed consent: A brief description of the study and its aims is then provided to the respondents. Their consent to participate in the study, record the interview, as well as use their information in the study reports, articles and thesis is obtained.

Engage in in-depth interviews: Interviews are carried out following predetermined topics of discussions

**Topic 1: Respondent's understanding of biodiversity offsets**.

The section seeks for the respondent's understanding and opinion of the biodiversity offset concept; opinions regarding the forms or models of biodiversity offsets implemented; and respondents' views regarding the potential of biodiversity offsets to attain no net loss. Probing questions include:

- What is your personal understanding of the 'biodiversity offsetting' concept?
- What should a biodiversity offset measure entail or constitute?
- What form of implementation can biodiversity offset measures take?
- What conservation activities can be implemented as biodiversity offsets?
- Can biodiversity offset measures achieve no net loss of biodiversity?
- How can no net loss be achieved while implementing biodiversity offsets?
- What are the reasons for your opinions above?
